# The safety and efficacy of nicotine replacement therapy in the intensive care unit: a randomised controlled pilot study

**DOI:** 10.1186/s13613-018-0399-1

**Published:** 2018-06-07

**Authors:** Ben de Jong, Anne Sophie Schuppers, Arriette Kruisdijk-Gerritsen, Maurits Erwin Leo Arbouw, Hubertus Laurentius Antonius van den Oever, Arthur R. H. van Zanten

**Affiliations:** 1 Department of Intensive Care Medicine, Gelderse Vallei Hospital, Willy Brandtlaan 10, 6716 RP Ede, The Netherlands; 2 Department of Intensive Care Medicine, Deventer Hospital, Nico Bolkesteinlaan 75, 7416 SE Deventer, The Netherlands; 3 Department of Clinical Pharmacy, Deventer Hospital, Nico Bolkesteinlaan 75, 7416 SE Deventer, The Netherlands

**Keywords:** Smoking, NNAL, Cotinine, Delirium, Agitation, Withdrawal

## Abstract

**Background:**

Studies evaluating nicotine replacement therapy (NRT) to prevent nicotine withdrawal symptoms in ICU patients have yielded conflicting results. We performed a randomised controlled double-blind pilot study to assess the safety and efficacy of NRT in critically ill patients. Mechanically ventilated patients admitted to two medical–surgical intensive care units and smoking more than 10 cigarettes per day before ICU admission were enrolled in this study. Participants were randomised to transdermal NRT (14 or 21 mg per day) or placebo until ICU discharge or day 30. Smoking status was confirmed by the biomarkers serum cotinine and urinary NNAL. The primary endpoint was 30-day mortality. Among secondary endpoints and post hoc endpoints, 90-day mortality, safety, time spent without delirium, sedation and coma, and patient destination at day 30 were addressed.

**Results:**

We enrolled 47 patients. No differences were found between NRT and control group patients concerning 30-day mortality (9.5 vs. 7.7%, *p* = 0.84) and 90-day mortality (14.3 vs. 19.2%, *p* = 0.67). The number of serious adverse events was comparable between groups (NRT: 4, control: 11, *p* = 0.13). At day 20, average time alive without delirium, sedation and coma was 16.6 days among NRT patients versus 12.6 days among control patients (*p* = 0.03). At day 30, more NRT group patients were discharged from the ICU or hospital compared with controls (*p* = 0.03).

**Conclusions:**

NRT did not affect mortality or the number of (serious) adverse events compared with placebo. Time alive without delirium, sedation and coma at day 20 in NRT patients was longer than in control patients. An adequately powered randomised controlled trial to further study safety and efficacy of NRT in ICU patients seems feasible and is warranted.

*Trial registration* ClinicalTrials.gov, number NCT01362959, registered 1 June 2011

**Electronic supplementary material:**

The online version of this article (10.1186/s13613-018-0399-1) contains supplementary material, which is available to authorized users.

## Background

Tobacco use is the leading cause of preventable deaths worldwide, killing 6 million people annually and reducing life expectancy by an average of 10 years [1, 2]. In 2015, 19% of the Dutch and 11% of the US population were daily smokers [3, 4]. For patients admitted to the intensive care unit (ICU), this is even higher and 25–47% are active smokers [5–7].

Active smokers admitted to an ICU, present more agitation, self-removal of devices, need for physical restraint and receive higher doses of sedatives, neuroleptics and analgesics [5]. Agitated behaviour might be a consequence of nicotine withdrawal, but may also be due to delirium or abstinence of concomitant alcohol and/or drug use. Until now it is unclear whether tobacco use confers higher risk of delirium during ICU stay [8].

Neuroadaptation leads to withdrawal symptoms in the abstinence of nicotine. Furthermore, smoking itself may also lead to a variety of pathological changes in organ systems (e.g. cardiovascular diseases) and affect multiple biological pathways, potentially increasing the risk for agitated behaviour [9, 10].

Nicotine replacement therapy (NRT) has been shown to reduce withdrawal symptoms in nicotine-dependent subjects who quit smoking [11, 12]. Research addressing the efficacy of NRT during critical illness shows conflicting results [13–22]. However, irrespective of its potential side effects and in the presence of inconsistent data on safety and efficacy, NRT is prescribed to prevent withdrawal symptoms or to treat agitated behaviour in smoking patients admitted to the ICU [23].

We designed a randomised controlled double-blind pilot study to assess the safety and efficacy of NRT in mechanically ventilated and actively smoking ICU patients.

## Methods

### Trial design

We performed a randomised, controlled, double-blind, pilot study between July 2012 and June 2016 that was approved by the Medical Research Ethics Committee (MREC) of the University Medical Centre of Utrecht and registered at ClinicalTrials.gov, number NCT01362959.

### Participants

Mechanically ventilated and actively smoking patients admitted to the medical–surgical ICU of two University-affiliated teaching hospitals, Gelderse Vallei Hospital (17 beds, GVH) and Deventer Hospital (12 beds, DH) were eligible for inclusion.

Exclusion criteria were: age < 18 years, last smoking > 72 h before inclusion, smoking ≤ 10 cigarettes/day, > 48 h after hospital admission admitted to the ICU, expected duration of mechanical ventilation ≤ 48 h, pregnant or breastfeeding, history of dementia or psychosis, neurologic disease on admission such as traumatic brain injury, intracranial haemorrhage, seizures, meningitis, encephalitis, intracranial tumour, cerebrovascular accident, NRT < 2 weeks before ICU admission, acute myocardial infarction, severe cardiac arrhythmia, unstable angina pectoris, generalised skin diseases, severe hearing deficiency, hypersensibility to nicotine or patches, insufficient Dutch language skills, imminent death or participation in another intervention study. Apart from regular exclusion criteria, we excluded factors interfering with the trial assessments and/or outcome or being a contraindication as mentioned in the Summary of Product Characteristics of the nicotine patches.

Written informed consent was obtained from all patients or their legal representatives. Retrospective written informed consent was obtained from the patient once mental capacity had regained.

### Procedures and interventions

After inclusion and before the start of study drugs, blood and urine samples were taken to determine serum cotinine levels (Immulite 2000 nicotine metabolite assay, Siemens Healthcare Diagnostics Limited) and urine 4-(methylnitrosamino)-1-(3-pyridyl)-1-butanol (NNAL) concentrations (analysis according to Xia et al. [24]) to confirm inclusion of actively smoking patients [25, 26]. For women of fertile age, a pregnancy test was performed. Patient characteristics were recorded including age, sex, weight, length, medical history including medication and allergies, Charlson Comorbidity Index (CCI), patient type (surgical/medical), Sequential Organ Failure Assessment (SOFA) score, Acute Physiology and Chronic Health Evaluation (APACHE) II and IV scores.

Treatment allocation was performed using restricted randomisation with blocks of four, with a 1:1 ratio to NRT or placebo. Patients were stratified to patient type (medical or surgical), nicotine exposure (< 21 cigarettes or ≥ 21 cigarettes/day) and study site (GVH or DH). After enrolment, healthcare workers not involved in ICU patient care performed randomisation. Randomisation codes were unknown to the investigators, ICU staff, patients and relatives.

Patients were treated with nicotine patches (Nicotinell^®^ TTS 20 and 30, Novartis Consumer Health) or similar size and shape, placebo patches (DuoDerm^®^ Extra Thin, ConvaTec), both subsequently covered by an opaque plaster (Fixomull^®^ stretch, BSN medical). Patches and covers were applied and replaced every 24 h until ICU discharge or day 30 by nurses not involved in ICU patient care, while ICU staff was not present. Patients smoking < 21 cigarettes/day received patches delivering 14 mg nicotine/24 h those smoking ≥ 21 cigarettes/day received patches delivering 21 mg nicotine/24 h.

Both hospitals involved in this study used sedation and agitation management protocols.

### Trial assessments

After inclusion, patients or legal representatives completed the Alcohol Use Disorders Identification Test (AUDIT) and the Fagerström Test of Nicotine Dependence (FTND) to assess alcohol consumption and nicotine dependency.

Part of routine ICU care was the daily assessment of the Richmond Agitation and Sedation Scale (RASS) score, Confusion Assessment Method for the ICU (CAM-ICU) score, Delirium Observation Scale (DOS) score, Behaviour Pain Scale (BPS) and the Numeric Rating Scale (NRS) at 08:00, 14:00 and 21:00. During the intervention period (day 1 until discharge from the ICU or day 30), hours of physical restraint was recorded as well as self-removal of catheters, self-extubations, nosocomial infections according to CDC-criteria [27], medication prescribed and hours of mechanical ventilation.

At day 30 and day 90, patient destination and survival status was confirmed by reviewing the electronic medical record (EMR) or by telephone interviews with the patient or their representatives.

Serious adverse events (SAEs) were reported by attending physicians to the principal investigator (PI). Potential relationships to the treatment were determined according to the definitions from the Guideline for Good Clinical Practice (version November 2016). All EMRs were screened for adverse events by the investigators.

### Outcomes

The primary endpoint was 30-day mortality. Secondary endpoints were 90-day mortality, ICU and in-hospital mortality, ICU and hospital length of stay (LOS), patient destination at day 30 and 90 (home, ICU, general hospital ward, nursing home, rehabilitation centre, deceased), hours with delirium assessed by the CAM-ICU or DOS-score, number of nosocomial infections, number of (serious) adverse events, number of self-removed catheters (i.e. arterial lines, peripheral and central venous catheters, nasogastric tubes, drains, urinary catheters), number of self-extubations, hours of physical restraint, hours without mechanical ventilation at day 30 (defined as persistent (non)invasive ventilation disconnection for at least 48 h), total dose of antipsychotics (i.e. haloperidol, olanzapine, quetiapine), RASS score and hours with RASS score outside the optimal range (score less than − 3 and greater than + 1).

A composite post hoc endpoint, reflecting return of normal brain function, was defined and assessed before unblinding the results comprising the number of hours alive without delirium, and without sedation (RASS ≥ 3) or coma. This endpoint was modified from a study addressing ICU sedation [28]. At day 10, 20 and 30, the average time spent with normal brain function between groups was compared.

### Statistical analysis

In order to evaluate the feasibility of an adequately powered trial to study the safety and efficacy of NRT among mechanically ventilated patients and actively smoking before ICU admission, a pilot study was conducted, assessing time investment, safety and effect size on a smaller scale. The initial sample size was set at 70, accepting lack of statistical power for the primary outcome parameter. No interim analyses were planned.

Results and baseline characteristics were described as medians with interquartile ranges (IQR), means with standard deviations (SD) or as numbers and percentages (%) when appropriate. Continuous variables were analysed using Student’s *t* test or the Mann–Whitney *U* test for normally distributed and non-normally distributed data, respectively. To analyse categorical variables Fisher’s exact or Chi-Square tests were used.

Kaplan–Meier survival plots were generated for the 30-day and 90-day mortality, and the survival curves were compared with log-rank tests.

The primary outcome parameter was subjected to logistic multivariate analysis, in which the stratification variables (patient type, nicotine dosage and study site) were included.

A *p* value < 0.05 was considered statistically significant.

Data were collected in a database using Microsoft Office Access 2007 and were analysed using IBM SPSS Statistics 22 on an intention-to-treat basis.

## Results

Between July 2012 and June 2016, eligibility was determined for 2715 admitted and mechanically ventilated ICU patients of whom 48 patients were enrolled: 27 patients at GVH and 21 at DH. As many patients met exclusion criteria, the inclusion rate was low. Therefore, after 4 years the study was stopped. The main reason for recruitment failure was elevated cardiac enzymes, often interpreted as a sign of instable angina pectoris or acute myocardial infarction, defined as exclusion criteria.

As one patient was excluded due to withdrawal of informed consent, 21 patients received NRT (of whom 62% a dose of 21 mg/day) and 26 patients received placebo. Baseline characteristics and serum cotinine and urinary NNAL concentrations were similar between groups and are shown in Table 1.

**Table 1:** Baseline characteristics of the patients

Characteristic	Nicotine replacement therapy (*N* = 21)	Control group (*N* = 26)
Age, years, mean (SD)	60.1 (10.55)	65. 2 (9.13)
Male sex, *n* (%)	12 (57)	16 (62)
BMI (m^2^/kg), mean (SD)	26.4 (6.75)	27.8 (5.95)
Charlson Comorbidity Index, median (IQR)	1 (0–1)	1 (0–2.25)
APACHE-II score, mean (SD)	19.0 (5.03)	21.1 (8.60)
APACHE-IV score, mean (SD)	69.7 (19.01)	75.9 (34.20)
Admission SOFA score, mean (SD)	6.5 (2.94)	6.9 (2.94)
Patient type (medical), *n* (%)	16 (76)	15 (58)
Smoking (cigarettes/day), median (IQR)	20 (12.5–27.5)	15 (14.5–25.0)
Alcohol (units/day), median (IQR)	2 (0–4)	2 (0–4)
FTND score, median (IQR)	5.5 (4–7.75)	5.0 (4–7)
AUDIT score, median (IQR)	5.5 (0.75–12)	5.0 (1–10.75)
Receiving nicotine 21 mg/day, *n* (%)	13 (62)	NA
Serum cotinine (ng/ml), median (IQR)	70.6 (25.8–110)	80.7 (37.5–126)
Urine NNAL (pg/ml), median (IQR)	117.6 (62.5–156.4)	177.9 (116.9–325.4)
Inclusion GVH, *n* (%)	12 (57)	14 (54)

*BMI* body mass index, *APACHE* Acute Physiology and Chronic Health Evaluation, *SOFA* Sequential Organ Failure Assessment Score, *FTND* Fagerström Test of Nicotine Dependence [0–4 (very) low, 5 medium, 6–7 high and 8–10 very high dependence], *AUDIT* Alcohol Use Disorders Identification Test (score ≥ 8 hazardous and harmful alcohol use), *NA* not applicable, *NNAL* 4-(methylnitrosamino)-1-(3-pyridyl)-1-butanol, *GVH* Gelderse Vallei Hospital

### Primary endpoint

**Fig. 1:**
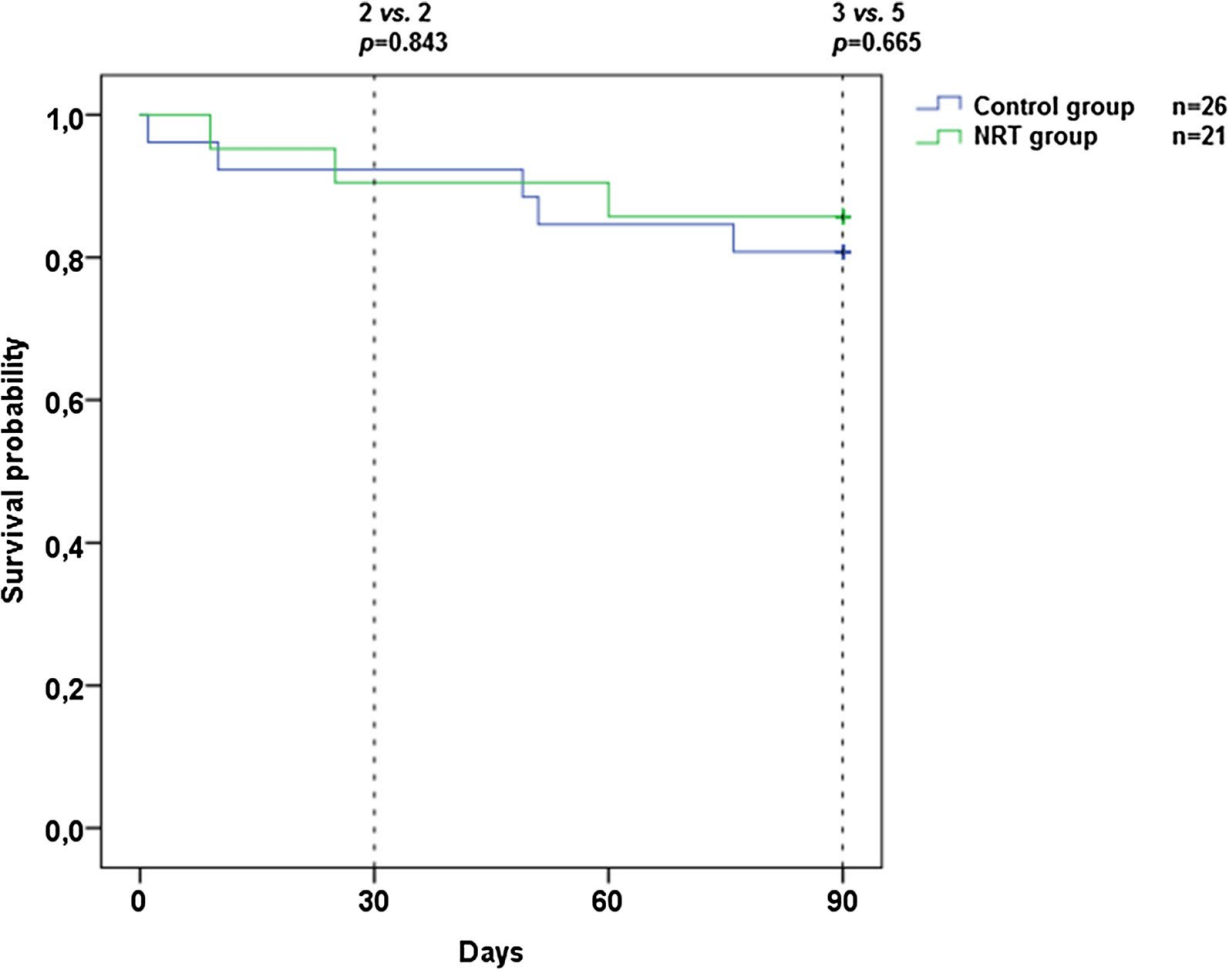
30-Day and 90-day mortality comparing nicotine replacement therapy and placebo group. *NRT* nicotine replacement therapy

The 30-day mortality rates for patients in the NRT and control groups were 9.5 and 7.7%, respectively (*p *= 0.84, Fig. 1). Multiple logistic regression analysis using the 3 predefined stratification groups as independent variables showed no effect of NRT on 30-day mortality [OR 0.96 (0.11–8.23)].

### Secondary clinical endpoints

**Fig. 2:**
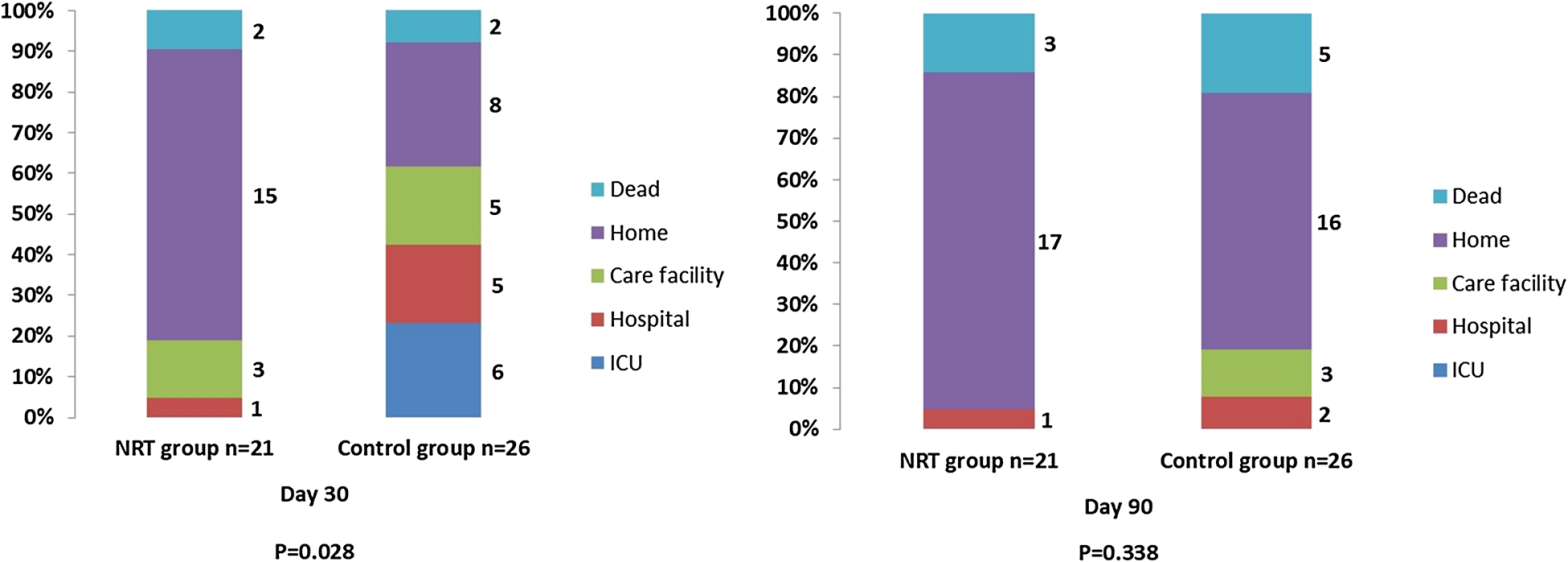
Patient destinations at day 30 and day 90 comparing nicotine replacement therapy and placebo group. Data are presented as numbers. *ICU* intensive care unit, *NRT* nicotine replacement therapy

The 90-day mortality rates for patients in the NRT and control groups were 14.3 and 19.2%, respectively (*p *= 0.67, Fig. 1). In the NRT group, fewer patients were still in the ICU or hospital at day 30 compared with the control group (1 vs. 11, *p *= 0.03), but not at day 90 (Fig. 2).

No differences were observed between NRT and control patients concerning in ICU and hospital LOS, hours without mechanical ventilation at day 30, total number of nosocomial infections, hours with delirium, time outside optimal sedation range, hours with physical restraint, number of self-removed devices and total dose of antipsychotics (Table 2).

**Table 2:** Secondary outcome parameters

Secondary outcome parameters	Nicotine replacement therapy (*N* = 21)	Control group (*N* = 26)	*p* value
ICU length of stay (h), median (IQR)			
Day 30	186 (127 to 278)	246 (88 to 694)	0.41
Day 90	186 (127 to 278)	246 (88 to 694)	0.392
Hospital length of stay (h), median (IQR)			
Day 30	313 (226 to 528)	408 (220 to 720)	0.356
Day 90	313 (226 to 528)	408 (220 to 885)	0.369
Mechanical ventilation-free hours at day 30, median (IQR)	559 (494 to 605)	515 (135 to 606)	0.152
Mechanical ventilation > 48 h, *n* (%)	17 (81)	20 (77)	–
Only non-invasive ventilation, *n* (%)	2 (10)	1 (4)	–
Nosocomial infections, *n* (%)	7 (24)	22 (76)	0.285
Hours with delirium, median (IQR)	8 (0 to 44)	16 (0 to 86)	0.152
RASS score, median (IQR)	− 1.0 (− 2.1 to − 0.2)	− 1.3 (− 2.3 to − 0.7)	0.266
Highest score	1 (0 to 1)	1 (0 to 1)	0.615
Lowest score	− 4 (− 5 to − 2.5)	− 5 (− 5 to − 4)	0.132
Outside optimal range (h)	40 (0 to 64)	48 (14 to 122)	0.202
Physical restraint (h), median (IQR)	12.0 (0 to 85.5)	44.5 (0 to 123)	0.417
Self-removed devices, *n* (%)			
Self-extubations	1 (20)	4 (80)	0.245
Catheters	24 (40)	36 (60)	0.886
Total dose of haloperidol (mg), median (IQR)	9 (0 to 24.5)	19.5 (3.25 to 31)	0.185
Serious adverse events, *n*	4	11	0.129
Adverse events, *n*			
Electrolyte disturbances	36	49	
Gastrointestinal	27	40	
Cardiovascular	16	43	
Arrhythmia	5	19	
Hypo-/hypertension	10	18	
Cardiac ischaemia	1	5	
Elevated cardiac enzymes	0	1	
Pulmonary	5	8	
Renal	1	6	
Others^a^	17	31	
Total adverse events, *n* (%)	102 (37)	177 (63)	0.096

*NRT* nicotine replacement therapy, *RASS* Richmond Agitation Sedation Scale, *mg* milligram, *h* hours, catheters are urinary and vascular catheters and nasogastric tubes

^a^Others: fever, fungal infection, sinusitis, allergic reaction, skin lesion, subcutaneous emphysema, thrombocytopenia, anaemia, pancytopenia, bleeding, hypo-/hyperthermia, hypothyroidism, ICU-acquired weakness, hypoventilation (hypercapnia), hemiplegia, anxiety

### Composite endpoint of normal brain function

**Fig. 3:**
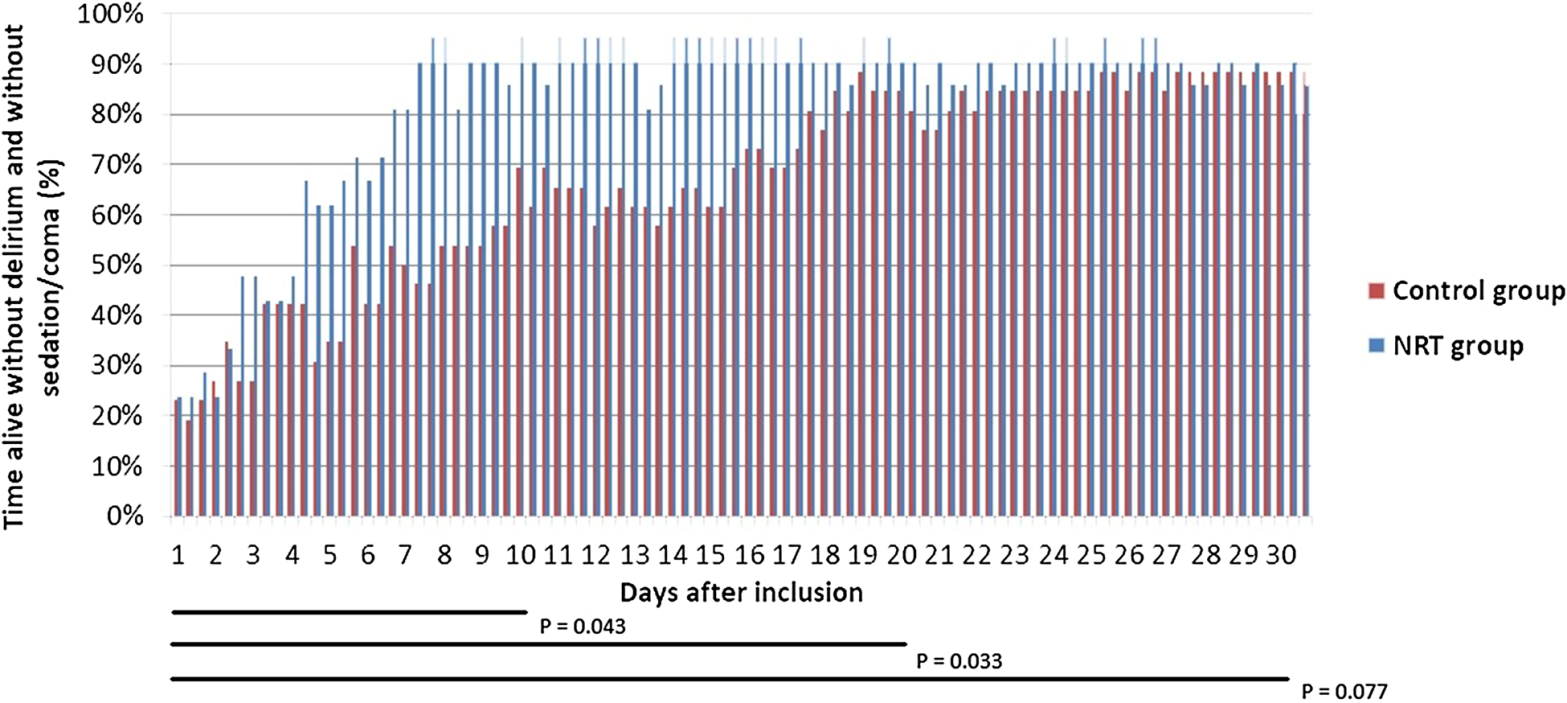
Patients alive without delirium and without sedation or coma comparing nicotine replacement therapy and placebo group. Data presented as the percentage of time patients were alive without delirium and without sedation or coma (RASS ≥ − 3). *NRT* nicotine replacement therapy

During the first 10 days, patients receiving NRT on average were alive without delirium and without sedation or coma for 160 h, versus 88 h in the placebo group. The difference in time with normal brain function was 72 h, which was statistically significant (*p* = 0.04). At day 20, this difference had increased to 104 h (i.e. > 4 days; *p* = 0.03). After 30 days, the difference was still 86 h, which was at that moment no longer significant (Fig. 3).

### Adverse events

In total, 15 SAEs were reported during the study period: 4 in the NRT group and 11 in the control group. In the NRT group, two patients died due to respiratory failure after extubation, but with do-not-reintubate orders. The other SAEs were reintubation due to respiratory insufficiency after extubation and a spontaneous haemothorax in a patient admitted with a pneumonia and congestive heart failure. No SAEs were thought to be related to NRT.

The number of adverse events in the NRT group compared with the control group was similar (102 vs. 177, *p* = 0.10, Table 2) as well as the number of cardiovascular adverse events (16 vs. 43).

## Discussion

This pilot randomised controlled trial provides data on the efficacy and safety of nicotine replacement therapy from mechanically ventilated patients, who were actively smoking before admission. Using biomarkers to distinguish active from passive smokers (serum cotinine ≥ 3.1 ng/ml or urinary NNAL ≥ 47.3 pg/ml), all patients were classified as actively smoking [25, 26].

We could not demonstrate differences between NRT and placebo groups with respect to mortality and (serious) adverse events. However, NRT was associated with more patients being discharged from the ICU or hospital at day 30. Moreover, patients receiving NRT spent more time alive without delirium and without sedation or coma during the first 20 days. This beneficial effect lost significance at day 30.

Available cohort and case–control studies assessing the role of NRT in supposed actively smoking ICU patients have shown conflicting results. Some studies suggested that NRT was associated with clinical benefits such as less agitation or decreased mortality, while others linked NRT to adverse effects such as increased ICU and hospital LOS, more delirium and need for physical restraining, increased duration of mechanical ventilation and more use of antipsychotics and even increased hospital mortality [13–19, 21]. Due to the retrospective design of all studies except one, selection bias and confounders may have influenced the reported results. In addition, varying inclusion and exclusion criteria were used, relevant differences in baseline characteristics were observed within some studies and heterogeneous populations were studied. Furthermore, smoking history obtained from patients or their legal representatives in general underestimates tobacco use in critically ill patients and there are no validated tools or questionnaires available to detect active smoking in ICU patients [25]. None of these studies used biomarkers such as cotinine or NNAL to confirm active smoking. Moreover, in case delirium was an outcome parameter, most studies did not use validated delirium instruments [13, 14, 19–21].

The only prospective pilot study that has addressed the effect of NRT in ICU patients (*n* = 40) demonstrated no effect of NRT on the use of sedatives and analgesics or ventilator free days compared with placebo [22]. However, there was a trend towards shorter ICU stay in the NRT group compared with the control group [4.5 (± 3.8) vs 7.0 (± 5.8) days, *p* = 0.08]. This study and our study suggested that NRT may lead to a shorter ICU and hospital LOS.

At study entry, brain dysfunction could be demonstrated in around 80% of patients in both groups. However, between admission and day 20, significantly more patients in the NRT group had regained normal brain function compared with the control group. This positive effect of NRT on brain function is in accordance with the time course of nicotine withdrawal symptoms, which peaks in the first week after abstinence and lasts for 2–4 weeks [29].

Our observation that the difference between groups disappeared after 3 weeks suggests that nicotine withdrawal symptoms may have vanished during the study period, and no beneficial effect of NRT on the withdrawal syndrome may be expected any longer after 3 weeks.

### Strengths and limitations

Proper patient selection is essential to evaluate the safety and efficacy of NRT in critically ill patients. In our study, the smoking status had to be confirmed by a questionnaire and had to be at a minimum of 10 cigarettes per day. The FTND classified patients as smokers with medium nicotine dependency. Serum cotinine or urinary NNAL confirmed that all patients were active smokers. Thorough patient selection with subsequent biomarker confirmation assured the inclusion of actively smoking patients and increased the validity of our study.

The main limitation of the study is the sample size with increased likelihood of type-1 or type-II errors. The small number of patients in the treatment arm (*n* = 21) precludes strong conclusions on safety and efficacy of NRT.

### Recommendations for further research on NRT in critically ill patients

When a similar design to our study would be used, a RCT with mortality as the primary endpoint would need a sample size of at least 6000 participants to detect a 20% difference between NRT and placebo (alpha 0.05, power 80%). With respect to feasibility, we suggest to use a composite endpoint of time alive without delirium, sedation or coma as the primary endpoint in future trials. This would necessitate inclusion of around 200 patients to detect a 48-h difference (alpha 0.05, power 80%). We suggest an intervention period as long as the withdrawal syndrome lasts (3–4 weeks).

An important reason for recruitment failure was the clinical indistinctness between myocardial infarction or ischaemia and increased cardiac enzymes for other reasons. When in doubt, attending physicians chose to exclude these patients. For future research, we advise clear definitions of cardiovascular events and no exclusion in the absence of a clear diagnosis by using 12-lead ECGs, repeated measurements of troponins, echocardiography to identify regional wall movement abnormalities and eventually angiography [30].

Although in our study clear effects of the present NRT could be demonstrated, in general the dosages of NRT to be used in future research and the route of administration are still unclear. In non-ICU patients, NRT is used to maintain some of the nicotine effects, but also to reduce the addiction potential by reducing the dosage and speed of delivery. Higher doses of NRT seem to be more effective in achieving smoking abstinence compared with lower doses [31]. However, during critical illness the main reason for prescribing NRT is to prevent or treat agitated behaviour. As ICU patients might have subcutaneous oedema or are treated with vasopressors, absorption of transdermal NRT may be compromised. Thus, nicotine inhalation or an oral or nasal spray and administration at a higher dose may better mimic smoking behaviour and potentially be more effective and could be considered in future studies [9, 11]. At present, there are no scientific data on the use of non-nicotine products to treat or prevent presumed nicotine withdrawal in critically ill patients (Additional file 1).

## Conclusions

Among patients, actively smoking before ICU admission and mechanically ventilated after ICU admission, transdermal nicotine replacement therapy had no effect on mortality compared with placebo, although our pilot study was underpowered to detect such difference. The numbers of (serious) adverse events between groups were comparable.

Patients in the nicotine replacement therapy group spent more time with normal brain function during the first 20 days after ICU admission compared with control patients. Moreover, at day 30, more patients in this group were discharged from the ICU or hospital compared with controls.

An adequately powered RCT to study safety and efficacy of NRT in ICU patients seems feasible and is warranted.

## Additional file

**Additional file 1.** Study protocol.

## Abbreviations

APACHE: Acute Physiology and Chronic Health Evaluation
AUDIT: Alcohol Use Disorders Identification Test
BPS: Behaviour Pain Scale
CAM-ICU: Confusion Assessment Method for the ICU
CCI: Charlson Comorbidity Index
DH: Deventer Hospital
DOS: Delirium Observation Scale
EMR: electronic medical record
FTND: Fagerström Test of Nicotine Dependence
GVH: Gelderse Vallei Hospital
ICU: intensive care unit
IQR: interquartile ranges
LOS: length of stay
MREC: Medical Research Ethics Committee
NNAL: 4-(methylnitrosamino)-1-(3-pyridyl)-1-butanol
NRS: Numeric Rating Scale
NRT: nicotine replacement therapy
PI: principal investigator
RASS: Richmond Agitation and Sedation Scale
RCT: randomised controlled trial
SAEs: serious adverse events
SD: standard deviations
SOFA: Sequential Organ Failure Assessment

## References

[1] World Health Organization (2011). WHO report on the global tobacco epidemic, 2011: warning about the dangers of tobacco.

[2] Jha P, Ramasundarahettige C, Landsman V (2013). 21st-Century hazards of smoking and benefits of cessation in the united states. N Engl J Med.

[3] Trend in roken volwassenen, 1990–2015. https://www.volksgezondheidenzorg.info/onderwerp/roken/cijfers-context/trends#definities. Accessed 11 Jan 2018.

[4] Burden of tobacco use in de U.S.: current cigarette smoking among U.S. adults aged 18 years and older. https://www.cdc.gov/tobacco/campaign/tips/resources/data/cigarette-smoking-in-united-states.html. Accessed 11 Jan 2018.

[5] Lucidarme O, Seguin A, Daubin C (2010). Nicotine withdrawal and agitation in ventilated critically ill patients. Crit Care.

[6] Van Rompaey B, Elseviers MM, Schuurmans MJ (2009). Risk factors for delirium in intensive care patients: a prospective cohort study. Crit Care.

[7] Moller AM, Pedersen T, Villebro N (2003). A study of the impact of long-term tobacco smoking on postoperative intensive care admission. Anaesthesia.

[8] Zaal IJ, Devlin JW, Peelen LM (2015). A systematic review of risk factors for delirium in the ICU. Crit Care Med.

[9] Benowitz NL (2010). Nicotine addiction. N Engl J Med.

[10] National Center for Chronic Disease Prevention and Health Promotion (US) office on smoking and health. The health consequences of smoking—50 years of progress: a report of the surgeon general. Atlanta (GA): Centers for Disease Control and Prevention (US); 2014. Chapter 5 (Nicotine) and Chapter 8 (Cardiovascular Diseases). 24455788

[11] Hatsukami DK, Stead LF, Gupta PC (2008). Tobacco addiction. Lancet.

[12] Fagerström KO, Schneider NG, Lunell E (1993). Effectiveness of nicotine patch and nicotine gum as individual versus combined treatment for tobacco withdrawal symptoms. Psychopharmacology.

[13] Mayer SA, Chong JY, Ridgway E (2001). Delirium from nicotine withdrawal in neuro-ICU patients. Neurology.

[14] Honisett TD (2001). Nicotine replacement therapy for smokers admitted to intensive care. Intensive Crit Care Nurs.

[15] Lee AH, Afessa B (2007). The association of nicotine replacement therapy with mortality in a medical intensive care unit. Crit Care Med.

[16] Paciullo CA, Short MR, Steinke DT (2009). Impact of nicotine replacement therapy on postoperative mortality following coronary artery bypass graft surgery. Ann Pharmacother.

[17] Panos NG, Tesoro EP, Kim KS (2010). Outcomes associated with transdermal nicotine replacement therapy in a neurosurgery intensive care unit. Am J Health Syst Pharm.

[18] Cartin-Ceba R, Warner DO, Hays JT (2011). Nicotine replacement therapy in critically ill patients: a prospective observational cohort study. Crit Care Med.

[19] Seder DB, Schmidt JM, Badjatia N (2011). Transdermal nicotine replacement therapy in cigarette smokers with acute subarachnoid hemorrhage. Neurocrit Care.

[20] Gillies MA, McKenzie CA, Whiteley C (2012). Safety of nicotine replacement therapy in critically ill smokers: a retrospective cohort study. Intensive Care Med.

[21] Kerr A, McVey JT, Wood AM (2016). Safety of nicotine replacement therapy in critically ill smokers: a retrospective cohort study. Anaesth Intensive Care.

[22] Pathak V, Rendon ISH, Lupu R (2013). Outcome of nicotine replacement therapy in patients admitted to ICU: a randomized controlled double-blind prospective pilot study. Respir Care.

[23] Kowalski M, Udy AA, McRobbie HJ (2016). Nicotine replacement therapy for agitation and delirium management in the intensive care unit: a systematic review of the literature. J Intensive Care.

[24] Xia Y, McGuffey JE, Bhattacharyya S (2005). Analysis of the tobacco-specific nitrosamine 4-(methylnitrosamino)-1-(3-pyridyl)-1-butanol in urine by extraction on a molecularly imprinted polymer column and liquid chromatography/atmospheric pressure ionization tandem mass spectrometry. Anal Chem.

[25] Hsieh SJ, Ware LB, Eisner MD (2011). Biomarkers increase detection of active smoking and secondhand smoke exposure in critically ill patients. Crit Care Med.

[26] Goniewicz ML, Eisner MD, Lazcano-Ponce E (2011). Comparison of urine cotinine and the tobacco-specific nitrosamine metabolite 4-(methylnitrosamino)-1-(3-pyridyl)-1-butanol (NNAL) and their ratio to discriminate active from passive smoking. Nicotine Tob Res.

[27] Horan TC, Andrus M, Duceck MA (2008). CDC/NHSN surveillance definition of health care-associated infection and criteria for specific types of infections in the acute care setting. Am J Infect Control.

[28] Pandharipande PP, Pun BT, Herr DL (2007). Effect of sedation with dexmedetomidine vs lorazepam on acute brain dysfunction in mechanically ventilated patients: the MENDS randomized controlled trial. JAMA.

[29] Hughes JR (2007). Effects of abstinence from tobacco: valid symptoms and time course. Nicotine Tob Res.

[30] Carroll I, Mount T, Atkinson D (2016). Myocardial infarction in intensive care units: a systematic review of diagnosis and treatment. J Intensive Care Soc.

[31] Stead LF, Perera R, Bullen C (2012). Nicotine replacement therapy for smoking cessation. Cochrane Database Syst Rev.

